# Induction of Th17 cell differentiation by B-1 cells

**DOI:** 10.3389/fimmu.2012.00281

**Published:** 2012-09-11

**Authors:** Yi Wang, Thomas L. Rothstein

**Affiliations:** ^1^Center for Oncology and Cell Biology, The Feinstein Institute for Medical ResearchManhasset, NY, USA; ^2^Department of Medicine, Hofstra North Shore-LIJ School of MedicineManhasset, NY, USA; ^3^Department of Molecular Medicine, Hofstra North Shore-LIJ School of MedicineManhasset, NY, USA

**Keywords:** B-1 cells, B-2 cells, Th17, CD86, CD44

## Abstract

B-1 cells constitute a unique B cell population with distinct ontogenic, phenotypic, and functional characteristics. Naïve, unmanipulated B-1 cells induce differentiation of CD4^+^ T cells to become pro-inflammatory Th17 cells whereas naïve B-2 cells do not. We examined the role of distinctly expressed surface membrane molecules in providing B-1 cells with Th17-differentiating function. Neither Mac-1, CD25, PD-L2 nor CD73 appeared to contribute to B-1 cell induction of Th17 differentiation. In contrast, we found that CD44 and CD86 are involved on the basis of studies with neutralizing antibodies and knock-out mice. Activation imparted to naïve B-2 cells the ability to induce Th17 differentiation and this was similarly partially interrupted by interfering with CD44 and CD86. Our findings suggest that CD44-OPN and B7 family members play important roles in the induction of Th17 cell differentiation by B cells.

## INTRODUCTION

CD4 T cells differentiate into separate helper, killer, regulatory, and inflammatory populations under the influence of various cytokines and cellular interactions that induce expression of specific transcription factors (Zhu et al., 2010). In particular, recent work has identified a unique Th17 cell population. These T cells are induced by TGFβ together with IL-6 or IL-21, plus IL-23 (Korn et al., 2007). Th17 cells produce and secrete one or more members of the IL-17 family of cytokines along with IL-22, and are characterized by expression of the transcription factors RORγt, RORα, and STAT3 (Korn et al., 2009). Th17 cells act as intermediaries that recruit other immune cells and produce inflammation that can be both advantageous in defense against infection and deleterious in fomenting autoimmunity (Awasthi and Kuchroo, 2009). Aberrant activity of Th17 cells plays a significant role in the pathogenesis of multiple inflammatory and autoimmune disorders, including SLE (Garrett-Sinha et al., 2008), allergic asthma (Oboki et al., 2008), inflammatory bowel disease (Fujino et al., 2003), and the murine model for multiple sclerosis, EAE (Komiyama et al., 2006). Thus, understanding the induction and regulation of Th17 cells is of paramount importance both from the standpoint of basic science and clinical medicine.

CD5^+^ B-1 cells constitute a special B cell population with distinct ontogenic, phenotypic, and functional characteristics (Baumgarth, 2011). B-1 cells derive from a unique progenitor and thus represent a separate lineage (Herzenberg and Tung, 2006; Montecino-Rodriguez et al., 2006). B-1 cells play a key role in immune system homeostasis by producing natural antibody that defends against common infectious pathogens and that disposes of molecular and cellular debris (Baumgarth et al., 2005; Binder and Silverman, 2005). Further, B-1 cells are thought to influence other elements of the immune system, through cytokine production and through direct interaction (O’Garra et al., 1992; Ghosn et al., 2008). Some of these activities may segregate with specific B-1 cell populations defined on the basis of surface antigen expression for Mac-1, CD25, CD73, and PD-L2, the latter of which has been shown to correlate with autoantibody production (Hastings et al., 2006; Zhong et al., 2007b, 2009; Ghosn et al., 2008; Tumang et al., 2011).

In previous work, we found that naïve peritoneal B-1 cells stimulated differentiation of CD4^+^ T cells to Th17 cells in an allogeneic co-culture situation (Hastings et al., 2006; Zhong et al., 2007b, 2009; Ghosn et al., 2008; Tumang et al., 2011). In direct contrast, co-culture of CD4^+^ T cells with naïve splenic B-2 cells did not yield Th17 cells but instead produced regulatory T (Treg) cells (Zhong et al., 2007a). This work was carried out under Treg promoting cytokine conditions. In the present study, we compared induction of Th17 cell differentiation produced by B-1 and B-2 cells in the presence of optimal cytokine conditions, and we determined the role that uniquely expressed B-1 cell surface antigens might play in this process.

## MATERIALS AND METHODS

### MICE

BALB/c, C57BL/6, CD44^– / –^, B7.1^– / –^, and B7.2^– / –^ double deficient mice (B7.1/2^– / –^; all knockout mice are on a C57BL/6 background) were obtained from The Jackson Laboratory. All mice were cared for and handled in accordance with NIH and institutional guidelines.

### ANTIBODIES AND REAGENTS

Fluorescently labeled antibodies (anti-CD4-FITC, anti-GL7-FITC, anti-CD21-FITC, anti-B220-FITC, anti-CD23-PE, anti-Mac1-PE, anti-CD80-PE, anti-CD86-PE, anti-CD44-PE, anti-Fas-PE, and anti-CD5-PE-Cy5) were obtained from BD Bioscience. Anti-OPN, anti-CD44, anti-CD80, anti-CD86, anti-IFNγ, anti-IL-4 antibodies and cytokines IL-2, IL-6, IL-21, IL-23, IL-27 were obtained from R&D systems. Anti-IL-17a-APC was obtained from eBioscience. LPS and PMA were obtained from Sigma-Aldrich. Ionomycin was obtained from Calbiochem. Brefeldin A was obtained from Enzo.

### CELL PREPARATION

Peritoneal washouts and spleens were obtained from 8–10-week-old mice, and then stained with fluorescence labeled antibodies. B cell populations (splenic follicular B cell: CD21^+^/CD23^+^; peritoneal B-1a: B220lo/Mac-1^+^/CD5^+^) were then purified by FACS using an Influx sorter (BD Bioscience). Flow cytometric analysis was performed using a LSR-II instrument (BD Bioscience). Naïve CD4^+^ T cells were purified from wild-type mice using the CD4^+^ T cell isolation kit (Miltenyi Biotec).

### *IN VITRO* Th17 CELL INDUCTION AND INTRACELLULAR CYTOKINE FLOW CYTOMETRY

Bead-enriched naïve CD4^+^ T cells were co-cultured at ratio of 2:1 with sort-purified, irradiated allogeneic B cells in 96-well round-bottom plates for 5 days in the presence of 10 μg/mL anti-INFγ, 10 μg/mL anti-IL-4, 3 ng/mL TGFβ, 50 ng/mL IL-6, and 20 ng/mL IL-23. Samples were stimulated with 50 ng/mL PMA and 800 ng/mL ionomycin and 10 μg/mL Brefeldin A for 5 h, before surface staining with combinations of antibodies against CD4 and intracellular cytokine staining with antibodies against IL-17A, and analyzed with a LSR II flow cytometer. All antibodies and staining buffers were purchased from eBioscience. Cell proliferation was measured as mean [3H]thymidine incorporation ± SD of duplicate wells.

## RESULTS

### B-1 CELLS, BUT NOT B-2 CELLS, INDUCE Th17 CELL DIFFERENTIATION UNDER OPTIMAL CYTOKINE CONDITIONS

**FIGURE 1:**
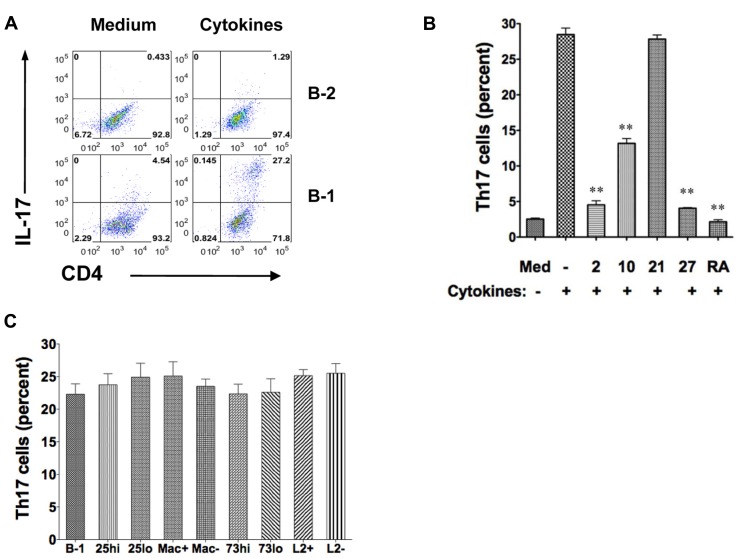
**B-1 cells, but not B-2 cells, induce Th17 cell differentiation under optimal cytokine conditions.** Sort-purified BALB/c peritoneal B-1 cells or splenic B-2 cells were co-cultured for 5 days at a 1:2 ratio with magnetic bead selected CD4^+^ T cells from C57BL/6 mice in the presence of medium only or Th17 polarizing cytokines. The percentages of alloactivated CD4^+^ T cells staining positively for intracellular IL-17 are shown. **(A)** Expression of intracellular IL-17 was assessed in CD4^+^ T cells by flow cytometry after stimulation by B-1 or B-2 cells in medium only or in the presence of Th17 polarizing cytokines TGFβ, IL-6, IL-23 plus anti-IL4 and anti-IFNγ (Cytokines). Results represent one of 3 comparable experiments. **(B)** Expression of intracellular IL-17 was assessed in CD4^+^ T cells by flow cytometry after stimulation by B-1 cells in medium only, or with Th17 polarizing cytokines (TGFβ, IL-6, IL-23 plus anti-IL4 and anti-IFNγ in the absence or presence of 10 ng/mL IL-2 (2), 10 ng/mL IL-10 (10), 10 ng/mL IL-21 (21), 10 ng/mL IL-27 (27) or 1 μM/mL retinoic acid (RA), as indicated. Mean values are shown along with lines indicating SEMs (*n* = 3). **(C)** Expression of intracellular IL-17 was assessed in CD4^+^ T cells exposed to Th17 polarizing cytokines and stimulated by B-1 cells or by sort-purified subpopulations of B-1 cells including CD25high (CD25^hi^) vs CD25low (CD25^lo^), Mac-1 positive (Mac^+^) vs Mac-1 negative (Mac^-^), CD73 high (CD73^hi^) vs CD73low (CD73^lo^), and, PD-L2 positive (L2^+^) vs PD-L2 negative (L2^-^). Mean values are shown along with lines indicating SEMs (*n* = 3). *t*-Test was performed to determine whether differences were significant. **p < 0.01; *p < 0.05.

Optimal conditions for Th17 cell differentiation include exposure of CD4^+^ T cells to TGFβ, IL-6, and IL-23, and blockade of IFNγ and IL-4. To more fully determine the differences between B-1 and B-2 cells in Th17 cell differentiation, we compared the capacity of irradiated, naïve peritoneal B-1 cells and irradiated, naïve splenic B-2 cells to induce Th17 cells in co-culture experiments under optimal conditions. B cells and T cells were allogeneically mismatched to more closely model what happens when T cells are activated by antigen presented in the context of MHC rather than by antibodies that recognize a TCR complex component. CD4^+^ T cells were examined for IL-17 expression by intracellular staining after 5 days. We found a marked difference between B-1 and B-2 cells (**Figure 1A**). Without added cytokines, B-1 cells induced a modest level of IL-17-containing T cells. With added cytokines, over one-fourth of T cells expressed intracellular IL-17. Notably, IL-17^+^ T cells generally expressed more CD4 than IL-17- T cells, presumably as a result of activation and enlargement. In direct contrast, B-2 cells without added cytokines did not induce Th17 cells and the presence of cytokines produced only a very small increase in Th17 cells to a level below that produced by B-1 cells in the absence of cytokines. Thus, under optimal cytokine conditions B-1 cells potently stimulate Th17 cell differentiation whereas B-2 cells completely fail to do so.

We examined the influence of additional cytokines on B-1 cell induction of Th17 cell differentiation (**Figure 1B**). We found that IL-2, IL-10, and IL-27 each inhibited Th17 cell differentiation (Laurence et al., 2007; Neufert et al., 2007). As expected, IL-21 had little effect in the presence of IL-6 and, also as expected, retinoic acid strongly blocked Th17 cell induction (Mucida et al., 2007).

We tested the role of several B-1 cell surface markers and the subpopulations defined by their expression in promoting Th17 cell differentiation. B-1 cells were divided into those that did or did not express Mac-1 (CD11b), those that did or did not express PD-L2, those that expressed high or low levels of CD25, and those that expressed high or low levels of CD73, as we have reported (Hastings et al., 2006; Zhong et al., 2007b; Tumang et al., 2011; Manuscript in preparation). Regardless of the subpopulation examined there was no alteration in B-1 cell stimulation of Th17 cell differentiation (**Figure 1C**), strongly suggesting that neither these molecules nor the subpopulations they define produce more or less induction of Th17 cells.

### CD86 CONTRIBUTES TO B-1 CELL-INDUCED Th17 CELL DIFFERENTIATION

**FIGURE 2:**
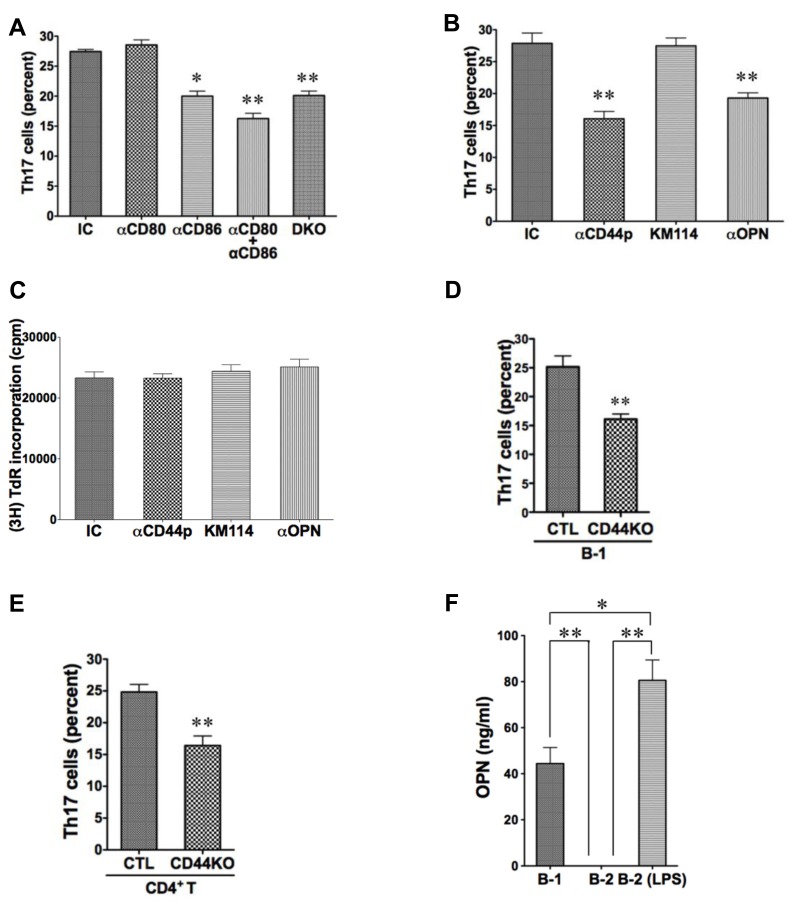
**CD44 and CD86 contribute to B-1 cell-induced Th17 cell differentiation. (A)** Sort-purified C57BL/6 peritoneal B-1 cells were co-cultured at a 1:2 ratio with magnetic bead selected CD4^+^ T cells from BALB/c mice in the presence of Th17 polarizing conditions with or without neutralizing antibodies directed against CD80 (αCD80) or CD86 (αCD86) or both, or with isotype control antibody (IC), as indicated. Sort-purified peritoneal B-1 cells from CD80/CD86 double knock-out mice on the C57BL/6 background (DKO) were co-cultured at a 1:2 ratio with magnetic bead selected CD4^+^ T cells from BALB/c mice. After 5 days expression of intracellular IL-17 was assessed by flow cytometry. Mean values are shown along with lines indicating SEMs (*n* = 3). **(B)** Sort-purified BALB/c B-1 cells were co-cultured at a 1:2 ratio with magnetic bead selected CD4^+^ T cells from C57BL/6 mice in the presence of Th17 polarizing conditions with either isotype control antibody (IC), polyclonal anti-CD44 antibody (αCD44p), KM114 monoclonal anti-CD44 antibody (KM114), or anti-OPN antibody (αOPN), each at 10 μg/mL. After 5 days expression of intracellular IL-17 was assessed by flow cytometry. Mean values are shown along with lines indicating SEMs (*n* = 3). **(C)** Comparable cultures as described in B were harvested at 3 days after addition of [^3^H]thymidine during the last 6 h of culture. Mean cpm values of triplicate cultures in three independent experiments are shown along lines indicating SEMs. **(D)** Sort-purified peritoneal B-1 cells from C57BL/6 (CTL) or CD44 KO mice were co-cultured at a 1:2 ratio with magnetic bead selected CD4^+^ T cells from BALB/c mice in Th17 polarizing conditions. After 5 days expression of intracellular IL-17 was assessed by flow cytometry. Mean values are shown along with lines indicating SEMs (*n* = 3). **(E)** Sort-purified peritoneal B-1 cells from BALB/c mice were co-cultured at a 1:2 ratio with magnetic bead selected CD4^+^ T cells from C57BL/6 (CTL) or CD44 KO mice in Th17 polarizing conditions. After 5 days expression of intracellular IL-17 was assessed by flow cytometry. Mean values are shown along with lines indicating SEMs (*n* = 3). **(F)** Sort-purified peritoneal B-1 cells and splenic B-2 cells were cultured for 5 days in medium, and sort-purified splenic B-2 cells were cultured for 5 days with 10 μg/mL LPS (B-2-LPS). Harvested supernatants were assayed for osteopontin (OPN). Mean values are shown along with lines indicating SEMs (*n* = 3). *t*-Test was performed to determine whether differences were significant. **p < 0.01; *p < 0.05.

Expression of CD80 and CD86 is elevated on B-1 as compared to B-2 cells, and blockade of CD86 eliminates B-1 cell-induced allogeneic stimulation of T cell proliferation (Zhong et al., 2007a). To investigate the potential role of CD80/CD86 costimulatory molecules in B-1 cell-induced Th17 cell differentiation, we added neutralizing anti-CD80 and anti-CD86 antibodies to B and T cells cultured as above and stained T cells for intracellular IL-17 5 days later. We found that anti-CD86 partially inhibited induction of Th17 cells, whereas anti-CD80 did not. However, blockade of both produced more inhibition than blockade of CD86 alone, suggesting a role for CD80 that may be masked by a strong stimulatory effect of intact CD86. To confirm the role of these co-stimulatory molecules we obtained B-1 cells from CD80^– / –^ CD86^– / –^ double knockout mice and verified inhibition of Th17 cell differentiation attributable to these B7 family members. (**Figure 2A**)

### CD44 CONTRIBUTES TO B-1 CELL-INDUCED Th17 CELL DIFFERENTIATION

Expression of CD44 is elevated on B-1 as compared to B-2 cells (Murphy et al., 1990). To investigate the potential role of CD44 in B-1 cell-induced Th17 cell differentiation, we added polyclonal neutralizing anti-CD44 antibody to B and T cells cultured as above and stained T cells for intracellular IL-17 5 days later. We found that anti-CD44 inhibited induction of Th17 cells by about half (**Figure 2B**). Because CD44 can bind both hyaluronic acid and osteopontin (Lesley et al., 1993; Naor et al., 1997; Ponta et al., 2003), we used additional neutralizing antibodies to determine which activity is involved in Th17 cell generation. We found that anti-osteopontin interfered with B-1 cell-induced Th17 cell differentiation, whereas an antibody (KM114) that solely interferes with HA binding to CD44 (Greyner et al., 2010) did not. Because CD44 can participate in cell–cell interaction (Siegelman et al., 1999), we examined allogeneic stimulation of T cell proliferation by B-1 cells in the presence or absence of these various antibodies to separate effects on T cell differentiation from general effects of B:T interaction. We found that none of the antibodies interfered with the ability of B-1 cells to drive T cell proliferation and thus did not prevent T cells from interacting with B cells (**Figure 2C**).

To confirm the role of CD44 we obtained B-1 cells from CD44^– / –^ knockout mice and verified inhibition of Th17 cell differentiation attributable to B-1 cell expression of this surface molecule (**Figure 2D**). At the same time we obtained CD4^+^ T cells from CD44^– / –^ knockout mice and verified inhibition of Th17 cell differentiation attributable to T cell CD44 expression (**Figure 2E**). Thus, CD44 participates specifically in B-1 cell stimulation of Th17 cell differentiation via its osteopontin binding activity, and CD44 on both B-1 cells and T cells is important.

### ACTIVATED B-2 CELLS STIMULATE Th17 CELL DIFFERENTIATION LIKE B-1 CELLS

**FIGURE 3:**
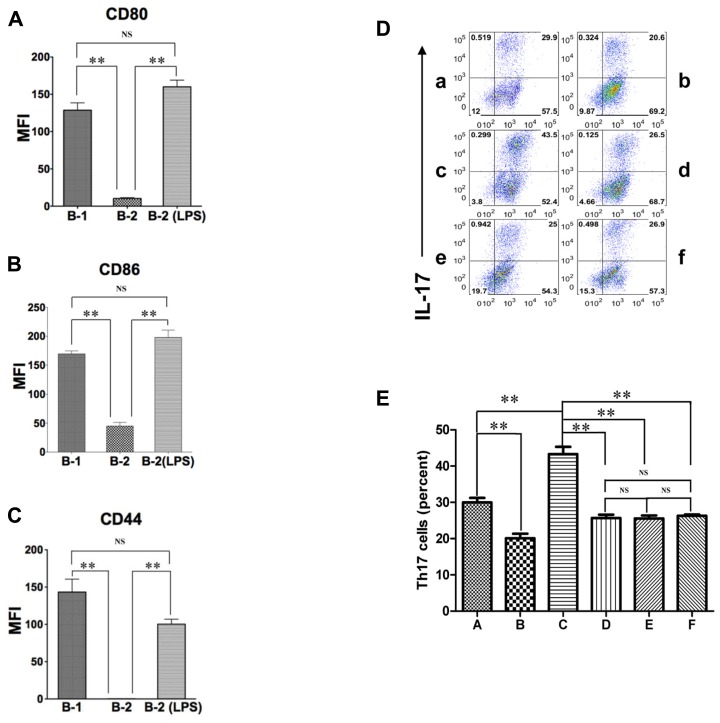
**Activated B-2 cells stimulate Th17 cell differentiation like B-1 cells. (A–C)** Peritoneal B-1 cells and splenic B-2 cells were evaluated for expression of CD80 **(A)**, CD86 **(B)**, and CD44 **(C)** by immunofluorescent staining and flow cytometry. Splenic B-2 cells were sort-purified and stimulated by 10 μg/mL LPS and then similarly evaluated. Mean values are shown along with lines indicating SEMs (*n* = 3). **(D,E)** Sort-purified BALB/c peritoneal B-1 cells **(a,b)** and splenic B-2 cells previously stimulated by 10 μg/mL LPS **(c–f)** were co-cultured at a 1:2 ratio with magnetic bead selected CD4^+^ T cells from CD57BL/6 mice in Th17 polarizing conditions in medium alone **(a,c)** or in the presence of anti-CD44 antibody **(d)**, anti-CD80 plus anti-CD86 antibody **(e)** or anti-osteopontin antibody **(b,f)**. After 5 days expression of intracellular IL-17 was assessed by flow cytometry. Representative results are shown in **(D)** and mean values (with horizontal lines indicating SEM) for five independent experiments are shown in **(E)**. In **(A,B,C,E)**, *t*-test was performed to determine whether differences were significant. **p < 0.01; *p < 0.05.

The expression of CD44, CD86, and CD80 is increased dramatically on B-2 cells by mitogenic stimulation (Murphy et al., 1990; Hathcock et al., 1994). Inasmuch as CD44, CD86, and CD80 are responsible, at least in part, for the ability of B-1 cells to induce Th17 cell differentiation, activated B-2 cells may acquire this ability as well. To test this possibility, we stimulated sort-purified naïve splenic B-2 cells with 10 μg/mL LPS for 48 h and then co-cultured activated B-2 cells with MHC-disparate CD4^+^ T cells in the presence of medium or optimal Th17 polarizing conditions. After 5 days T cells were stained for intracellular IL-17. We found that activated B-2 cells strongly induced Th17 cell differentiation, even exceeding the induction produced by naïve B-1 cells (**Figure 2F**) and highly expressed CD80, CD86 and CD44 (**Figures 3A–C**). To evaluate the role of LPS-stimulated expression of CD44 and B7 family members in this process, we co-cultured activated B-2 cells with naïve T cells in the presence of neutralizing antibodies directed against CD44, CD80/86, and osteopontin. We found that each of these antibodies produced an approximately 40% decline in Th17 cell induction (**Figure 3D,E**). Thus, naïve B-1 cells are capable of inducing Th17 cell differentiation as are activated (but not naïve) B-2 cells and key surface molecules including CD44 (osteopontin), CD86 and CD80 are involved in the induction of Th17 cells by B cells whether acquired natively (B-1 cells) or as a result of activation (B-2 cells).

## DISCUSSION

Antigen-presenting cells (APC) are principal architects of the immune response by orchestrating T cell priming and differentiation. Much effort has been focused on understanding the role and function of dendritic cells (DC) in these processes. Although B cells are also considered professional APC, previous work has focused primarily on their role as terminal effectors of antibody production dependent on the help of DC-primed T cells, whereas their role in T cell priming, differentiation, and tolerance has been controversial.

Recent evidence has highlighted the role of Treg cells in the induction and maintenance of peripheral tolerance and, conversely, the role of Th17 cells as effectors of inflammatory responses. Besides naturally occurring Treg (nTreg) cells, induced Treg (iTreg) cells can be converted from naïve T cells in the presence of TGFβ. Such conversion also requires APC-derived costimulatory signals in addition to TCR engagement. We questioned whether B cells function as APC in the generation of Th17 cells and what surface molecules might be involved. We focused on B-1 cells, a distinct B cell subset that produces protective natural antibody and that differs from B-2 cells in many respects including lineage, location, gene expression, antibody repertoire, proliferative responses, and immunoglobulin secretion.

In recent years a role for B cells has been delineated in a number of autoimmune and inflammatory diseases, which has led to successful application of B cell depletion therapies in some (Perosa et al., 2010). The results presented here emphasize the capacity of naïve B-1 cells and activated B-2 cells to induce differentiation of IL-17 expressing pro-inflammatory Th17 cells, raising the possibility that participation in Th17 cell induction represents a mechanism by which B cells may contribute to autoimmune and inflammatory disease. Our results further indicate that osteopontin-binding CD44 plays a major role in the pathway from B cells to Th17 cells *in vitro*, which fits well with recent results indicating a role for CD44/osteopontin in promoting Th17 cell differentiation *in vivo* (Guan et al., 2011). This latter work did not determine how T cells were stimulated to undergo Th17 cell differentiation, which the work presented here suggests may come about through B cell:T cell interaction. B-1 cells are known to produce IL-10 (O’Garra et al., 1992) and the influence of CD44/osteopontin may result, in part, from inhibition of immunosuppressive IL-10 secretion (Murugaiyan et al., 2008) that allows for more robust T cell stimulation. This mechanism may also contribute to disease which has been shown in mouse and human to be associated with osteopontin (Iizuka et al., 1998; Forton et al., 2002; Jansson et al., 2002; Yumoto et al., 2002; Xu et al., 2005).

In summary, our findings suggest that CD44/osteopontin and CD86/CD80 costimulatory molecules play a crucial role in the induction of Th17 cells by B cells. These data show that B-1 cells and activated B-2 cells can direct and regulate the inflammatory response through Th17 induction. To the extent that they do so, B cell depletion therapy may reset the balance between effector and regulatory populations of T cells as well as B cells.

## Conflict of Interest Statement

The authors declare that the research was conducted in the absence of any commercial or financial relationships that could be construed as a potential conflict of interest.

## Abbreviations

CTLA: cytotoxic T lymphocyte-associated antigen
HA: hyaluronic acid
OPN: osteopontin
